# Role of natural products towards the SARS-CoV-2: A critical review

**DOI:** 10.1016/j.amsu.2022.104062

**Published:** 2022-07-02

**Authors:** Kannan Raman, Kalirajan Rajagopal, Fahadul Islam, Manish Dhawan, Saikat Mitra, Baliwada Aparna, Potlapati Varakumar, Gowramma Byran, Om Prakash Choudhary, Talha Bin Emran

**Affiliations:** a Department of Pharmaceutical Chemistry, JSS College of Pharmacy (JSS Academy of Higher Education & Research), Ooty, The Nilgiris, Tamil Nadu, India; b Department of Pharmacy, Faculty of Allied Health Sciences, Daffodil International University Dhaka, 1207, Bangladesh; c Department of Microbiology, Punjab Agricultural University, Ludhiana, 141004, Punjab, India; d Trafford College, Altrincham, Manchester, WA145PQ, UK; e Department of Pharmacy, Faculty of Pharmacy, University of Dhaka, Dhaka, 1000, Bangladesh; f Department of Veterinary Anatomy and Histology, College of Veterinary Sciences and Animal Husbandry, Central Agricultural University (I), Selesih, Aizawl, India; g Department of Pharmacy, BGC Trust University Bangladesh, Chittagong, 4381, Bangladesh

**Keywords:** SARS-CoV-2, COVID-19, Natural compounds, Herbal medicine, ACE2

## Abstract

Despite the fact that various therapeutic compounds are being investigated, there is still a scarcity of effective and reliable therapeutic regimens to treat COVID-19. Ever since the COVID-19 pandemic, a diversity of traditional herbal treatments has been investigated to cure infected people, either alone or in conjunction with mainstream pharmaceuticals, with encouraging outcomes. In this article, we look at the latest research on the usage of natural products to alleviate the severity of COVID-19. To determine the activity of the natural products, act against SARS-CoV-2 to various targets like M^pro^, ACE-II, papain-like, chymotrypsin-like proteases, and some antiviral targets. The processes underlying this preventative or therapeutic action are also examined. We used PubMed, Scopus, Google Scholar, and the WHO site to perform our review. The *anti*-SARS-CoV-2 impacts of various herbal extracts and purified compounds may be mediated via direct prevention of viral replication or entrance. Interestingly, certain items might avert SARS-CoV-2 from infecting human cells by blocking the ACE-2 protein or the serine protease TMPRRS2. Natural products have also been stated to suppress proteins intricate in the virus life cycle, like papain-like and chymotrypsin-like proteases. To conclude, natural products can be used alone or in combination as remedies or treatments for COVID-19. In addition, their compositions may provide insight into the development of effective and reliable antiviral drugs.

## Highlights

• The COVID-19 pandemic caused by a novel coronavirus SARS-CoV-2, resulted in thousands of deaths across the world.
• Globally, numerous studies and researchers have recently started fighting this virus.
• In this article, we look at the latest research on the usage of natural products to alleviate the severity of COVID-19.
• The *anti*-SARS-CoV-2 impacts of various herbal extracts and purified compounds may be mediated via direct prevention of viral replication or entrance.

## Abbreviation

SARS-CoV-2: severe acute respiratory syndrome coronavirus 2
WHO: World Health Organization
ACE-2: Angiotensin-Converting Enzyme 2
tmprss2: Transmembrane serine protease 2
3CLpro: 3CL protease inhibitor
RNA: Ribonucleic acid
ER: Endoplasmic reticulum
FERT: Fluorescence Resonance Energy Transfer
IC_50_: half-maximal inhibitory concentration
SARS-CoV: severe acute respiratory syndrome coronavirus
MERS: Middle East respiratory syndrome

## 1 Introduction

The COVID-19 pandemic, caused by a novel coronavirus SARS-CoV-2, resulted in thousands of deaths across the world [1]. In addition, to the significant death toll, a substantial number of negative impacts have been reported on the health care infrastructure and economic structures of many nations. Additionally, the evolution of SARS-CoV-2 has resulted in the emergence of several different strains of SARS-CoV-2, resulting in consecutive waves of COVID-19 cases among various countries [2]. In context to the negative repercussions of the COVID-19 pandemic, several vaccines and therapeutic regimens have been developed to combat the negative impacts. To cure COVID-19, certain therapeutic options have been recommended, including nucleoside analogs, Remdesivir, anti-inflammatory medications, or Lopinavir/Ritonavir. Over 200 clinical studies have been filed in clinical trials. Gov, some of which are evaluating these medicines or others. There is no doubt that a substantial advancement has been made in the development of therapeutic regimens, but still, there are no FDA-approved therapies. Nonetheless, the scientific efficacy of these medicines in the action of corona infection is unknown [3]. Since the beginning of the COVID-19 contagion, traditional natural remedies have been employed in China. Furthermore, traditional medicines were found to help 90% of the 214 individuals who were administered recover. In addition, certain traditional herbal treatments protected healthy people from SARS-CoV-2 infections and enhanced the care of individuals with moderate or severe signs. In China's Zhejiang Province, similar encouraging outcomes were observed. Shu Feng Jie Du and Lianhuaqingwen, two traditional Chinese remedies, have been preferred due to their effectiveness over past influenza A (H1N1) or SARS-CoV infections [4]. Traditional medicines were incorporated into the therapies for the management and control of COVID-19 by a panel of specialists from Wuhan University's Zhongnan Hospital. Several plant-basedtherapeutic regimens have been suggested to alleviate the COVID-19 symptoms. Furthermore, to treat the condition, the experts advise using a variety of herbal blends as per the stage of the disorder [5,6]. Various medicinal plants such as *Allium sativum, Camellia sinensis, Zingiber officinale, Nigella sativa, Hypericum perforatum, Glycyrrhiza glabra*, and *Scutellaria baicalensis* have been exploited to boost the immunological response of patients infected with SARS-CoV-2 [7]. Different forms of terpenoids appear to have potential effects in inhibiting viral replication and might be used in future investigations. Furthermore, alkaloid compounds, including homoharringtonine, lycorine, and emetine, show potent *anti*-coronavirus properties. Different coronavirus targets, such as S-protein (emodin, baicalin) and viral enzymes replication, such as 3CLpro (Iguesterin), PL^pro^ (Cryptotanshinone), helicase (Silvestrol), and RdRp, can be inhibited by natural products (Sotetsuflavone). Natural products can be used as preventative and therapeutic agents in the battle against coronavirus, according to prior research [[7], [8], [9]].

Hence, this review looks at how herbal-based traditional drugs and natural products (NPs) might be used to manage and cure COVID-19 disease with their probable mode of action against the targets (Table 1).

**Table 1:** Natural sources examined towards SARS-CoV-2.

SL. No.	Plant family	Source	Strains	Assay name	Dose	Effects	References
1.	*Alnus japonica*	Hirsutenone	In vitro SARS-CoV- Plpro	FRET	0–200 μM	A dosage-based suppression of SARS-CoV-PLpro action	[15]
2.	*Cibotium barometz*	Ethanol and methanol Extracts	In vitro SARS-CoV virus propagated in Vero E6 cells	ELISA and FRET	0, 25, 50, 100 and 200 μg/ml	Both extracts repressed the SARS-CoV repetition at 25 and 200 mg/ml concentrations	[16]
3.	*Cullen corylifolium*	Psoralidin	In vitro SARS-CoV-PLpro	Fluorogenic	0–100 μM	Suppression of SARS-CoV PLpro in a dosage-based Manner	[17]
4.	*Ecklonia cava*	Ethanol extract of Dieckol	In vitro SARS-CoV- 3CL (pro)	FRET	0–200 μM	Suppression of SARS-CoV- 3CL (pro) Action	[18]
5.	*Scutellariabaicalensis Georgi*	Scutellarein	In vitro	FRET	0.01–10 μM	Suppression of SARS-CoV helicase through disturbing the ATPase action	[19]
6.	*Tribulus terrestris*	Methanol extract of Terrestrimine	In vitro SARS-CoVPLpro	Fluorogen	1, 10, 100, 1000 μM	Suppression of SARS-CoV – Plpro through IC_50_ = 15.8 ± 0.6 μM	[20]
7.	*Tribulus terrestris*	Methanol extract of Terrestrimine	In vitro SARS-CoV-2 virus which is proliferated in E6 virus	Cytopathic impact suppression	0–600 μg/ml/72 h	Suppression of SARS-CoV-2 repetition	[21]
8.	*Rheum* sp.	Emodin	In vitro Vero cells	Luciferase assay	0, 10, 50, 100, 200 & 400 μM	Obstruction of binding SARS-CoV S receptor and ACE2	[22]
9.	*Polygonum* sp.	Emodin	In vitro Vero cells	Luciferase assay	0, 10, 50, 100, 200 & 400 μM	Slight active concentration	[22]
10.	*Angelica keiskei*	Xanthoangelol E (Ethanol extract)	In vitro SARS-CoV- PLpro	FRET	0, 12, 5, 25, 50 μM	A dosage dependent suppression of SARS-CoV-PLpro activity	[23]
11.	*Angelica keiskei*	Xanthoangelol E (Ethanol extract)	In vitro SARS-CoV-3CL (pro)	FRET	0, 12, 5, 25, 50 μM	A dosage dependent suppression of SARS-CoV-3CLpro activity	[23]

## 2 Natural products against SARS-COV-2

SARS-CoV-2 a member of the beta genus of the Coronaviridae family's Nidovirales order. Khan et al. describe SARS-CoV-2 as an enclosed, single (+) stranded RNA with symmetric helical nucleocapsid [10]. The virus contains twenty proteins, comprising four protein aggregates, namely, S denotes spike, E is the envelope, M represents membrane, and N is the nucleocapsid, as well as many nonstructural proteins like RNA-dependent RNA polymerase (RdRp), coronavirus main protease (3CL^pro^), and papain-like protease (PL^pro^) [11].

SARS-CoV-2's ability to bind to human and bat cells and proliferate was discovered to be dependent on the angiotensin-converting enzyme II (ACE2) enzyme. Viruses infect host cells by binding with the ACE2 receptors via a spike protein-receptor binding domain's protein-binding motif (RBD) [12]. The C-terminal S2 subunit of the spike protein (which is required for virus-cell membrane fusion) will shift shape as a result of this interaction. The host cellular-type 2II transmembrane serine protease TMPRSS2 subsequently proteolytically processes the complex S protein-ACE2, resulting in ACE2 breakdown and hence viral entrance through into host organism [13]. After access and uncoating, genomic RNA is transcribed to 2 polyproteins (pp1a and b), which were sliced by proteases to produce 15–16 nonstructural receptors. The nonstructural protein causes the cell membranes to reorganize, resulting in the formation of double-membrane vesicles. On the one hand, genomic RNA is translated into sub-genomic RNA, which results in structural (spike, envelope, membrane, and nucleocapsid) and auxiliary proteins being produced. Before being released through the secretory pathway, virions were eventually joined together in the ERGolgi intermediate complex. SARS-CoV-2 is genetically and clinically similar to other beta-genus coronaviruses, such as SARS-CoV and NL63. To enter the body, both viruses need to make contact with the ACE2 receptor. Nevertheless, changes in the sequence of S protein as well as the assembly of the protein binding area have been documented across different strains [14]. SARS-CoV-2 and SARS-CoV, on the other side, have a higher nucleotide homology, as well as a significant homology (95–100%) among the proteins of two strains. SARS-CoV-2 and SARS-CoV had 99, 90% comparable S2 and N proteins, respectively.

## 3 Antiviral effects

### 3.1 Antiviral effects of NPs

Investigated the anti-inflammatory and suppressing impacts [21] of a Chinese herbal-based concoction known as Lianhuaqingwen (a combination of 11 traditional therapeutic plants and a mineral) towards SARS-CoV-2 (gypsum and menthol drug) as shown in Table 2.

**Table 2:** Traditional applications of therapeutic species and combinations with probable antivirus impacts.

SL. No.	Plant family	Traditional applications	References
1.	*Alnus japonica*	Tumor, Blood, and lymphatic disease	[24]
2.	*Ecklonia cava*	Infections, Asthma, Diabetes and Tumor	[25,26]
3.	*Onopordumacanthium* L.	Hypertension, Homeostasis, Microbialinfections	[27]
4.	*Cullen corylifolium*	Eczema, Pollakiuria, Asthma	[28]
5.	*EphedraeHerba*	Inflammations andFever	[29]
6.	*Tribulus terrestris* L.	CardiovascularandHormonaldisorders	[30]
7.	*DioscoreapolystachyaTurcz.*	Liver disorders and Diabetes	[31]
8.	*Salvia miltiorrhiza Bunge*	Inflammations Cardiovascular and circulatory disorders	[32]
9.	*Senna tora*	Constipation and Liver disorders	[33]
10.	*Quercus infectoria G.*	Dysentery, Infections	[34]

Lianhuaqingwen was used to reduce fever, coughing, tiredness, influenza, bronchitis, asthma, and the early stages of measles for centuries involved in phase 2 clinical research in the United States. The Chinese National Health Commission suggested this herbal combo to cure or manage COVID-19. Cytopathic impact suppression and plaque reduction assays were used to evaluate antiviral efficacy in Vero E6 cells; with the IC_50_value of 411.2 μg/ml, the medicinal mixture decreased SARS-CoV-2 repetition in a daily dosage way. Moreover, in a dose-dependent way, the mix was capable of decreasing the expression of pro-inflammatory [21] cytokines. These findings are intriguing because cytokine storm has now been identified as among COVID-19's deadly consequences. In a recent investigation, seven compounds (arctiin, gallic acid, secoxyloganin, forsythoside A, isoliquiritigenin, rutin, and kaempferol) had IC_50_ values ranging from 4.9 0.1 M (kaempferol) to 47.8 1.5 M (kaempferol) were found to exhibit considerable antiviral activity (secoxyloganin) [35]. Four COVID-19 patients were utilized in the treatment of lopinavir/ritonavir and arbidol plus capsules of ShufengJiedu, [36]. Three individuals were determined to be COVID-19 negative after therapy and had substantial improvements in the signs. Another research of 132 COVID-19 individuals in China found that traditional Chinese medicine (TCM) was employed in about 92% of cases. The optimal beneficial method, according to the research, was a mixture of Kaletra and conventional medicine. Using in silico techniques, theaflavin might be employed as an essential *anti*-SARS-CoV-2 medicine. Furthermore, theaflavin displayed enticing docking affinities inside the corona RdRp catalytic region. Nonetheless, because they are not absorbed in significant numbers, their bioavailability may restrict their utility, and the theaflavin skeleton has been proven to be resistant to microbial breakdown. Single observational studies on the effectiveness of herbal drugs toward SARS and H1N1 influenza viruses have led to the conclusion that therapeutic species, normally employed as herbal formula, might be a promising defensive strategy for higher-risk groups. *Glycyrrhiza glabra* L., *Astragalus mongholicus* Bunge, *Atractylodeslancea* (Thunb.) DC., *Saposhnikoviadivaricata* (Turcz. ex Ledeb.) Schischk., *Atractylodesmacrocephala*Koidz., *Lonicera japonica*Thunb., *Lonicera japonica*Thunb. and Fors. These are all the constituents of Yupingfeng powder, a traditional Chinese medication [37]. The ethanol extraction of *Sambucus javanica*, Fukuoka stem, on either hand, showed significant anti-human coronavirus NL63 activity, with IC_50_ values varying from 1.17 to 15.75 μg/ml. The extract reduced virus output, plaque development, and virus attachment considerably. Three of its main phenolic acids (caffeic, gallic acid, and chlorogenic) have also been demonstrated to suppress NL63 proliferation and virus adherence. The most powerful phenolic acid was caffeic acid. Phenolic acids are distinguished by their capacity to be metabolized by the microbiota, which increases their bioavailability. Furthermore, the length of the alkyl chain could boost its antiviral potency [38]. Nevertheless, due to limited absorption and stability in alkaline and neutral conditions, their efficiency is still debatable, which may limit their use in pure form. As a result, the clinical value of phenolics as *anti*-SARS-CoV-2 medicines is still questionable, as their bioavailability, delivery methods, and effective dosages need to be investigated further utilizing in vivo studies.

Using a cell-based test, an investigation of 200 Chinese herb excerpts for its *anti*-SARS-CoV efficacy. Six extracts were proven to greatly suppress SARS-CoV development and multiplication. The IC_50_ concentrations ranged from 25 to 200 mg/ml. The study found that isolates from the tuber of *Dioscorea polystachya* Turcz. as well as the rhizome of *Cibotium barometz* inhibited SARS-CoV-3CL protease at IC_50_s of 39 and 44 mg/ml, to use the FRET assay [16].

**Fig. 1:**
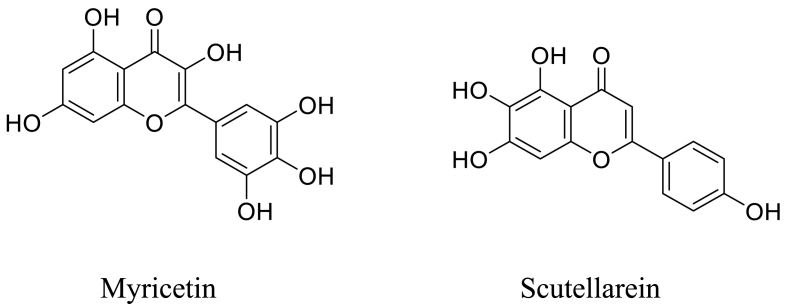
Natural-based compound that performs as viral helicase action.

SARS-CoV helicase remained an aim of innovative antiviral medicines that are relevant as a critical receptor for SARS-CoV gene replication. The repressive impacts of 64 natural substances derived from 15 medicinal species against the SARS-CoV helicase were tested. SARS-CoV helicase activity was considerably suppressed by myricetin and scutellarein (Fig. 1). At 10 μM, myricetin and scutellarein were capable of blocking 90% of the SARS-CoV helicase's ATPase activity. As a result, myricetin and scutellarein have been suggested as potential future *anti*-SARS medications [19].

Antiviral medicines are being developed to aimat receptors intricate in the SARS-CoV-2 shelf life. Consequently, substances and/or goods that block these receptors might be utilized to cure or avert disease perhaps stop SARS-CoV-2 impurities from spreading (Table 1).

### 3.2 Role of NPs as ACE2-blockers

Because the SARS-CoV-2 spike protein binds to host proteins, the viral genome can penetrate to host cells. Phylogenetic study ACE2 structural study and crucial site, various animals Cats, pigeons, and lambs, for example, were projected to be significant virus intermediary hosts [39]. Hoffmann et al. found thatACE2 receptor was employed through the immune system. Coronavirus is capable of infecting human cells. Furthermore, they stated that TMPRSS2 antagonists could be a capable dealing choice. The TMPRSS2 pierces both ACE2 as well as the S protein [36]. Ortega et al. employed in silico techniques to better understand the link between SARS-CoV-2 Spike protein and ACE2 receptor alterations. They revealed spike proteins have a sophisticated affinity for human ACE2 than the Bat-CoV spike receptor^37^. The ACE2 receptor could be the major “bridge” for use through SARS-CoV-2 to transfer across humans, according to this research. At the same time, SARS-CoV and SARS-CoV-2 RBD of spikes glycoprotein have 72% structural similarity. Chen et al. established that coronavirus RBD had considerable overlap with ACE2. ACE2 antagonists were hypothesized to suppress coronavirus infection via altering the RBD binding domain indirectly [11]. Authors have discovered that the S-protein of SARS-CoV-2 had a larger affinity for ACE2 compared to the SARS-CoV virus [40]. The study has shown that one of the methods exploited by new effective *anti*-SARS medications is early inhibition of SARS-CoV using ACE2 antagonists. Despite the use of ACE2 antagonists, three recent kinds of coronavirus research found that hypertension and diabetic Mellitus greatly increased the likelihood of coronavirus infections. The elevation of ACE2-by-ACE2 antagonists, ACE II receptor blockers, and ibuprofen supports the immediate need to employ and/or find alternate ACE2 blockers. As a result, medicinal plant-derived product lines or NPs that selectively hinder the ACE2 protein without hindering enzyme action could be effective at preventing and treating SARSCoV-2 transmission in humans without raising ACE2 representation inpatient role and thus raising the hazard of COVID-19 infection [41].

**Fig. 2:**
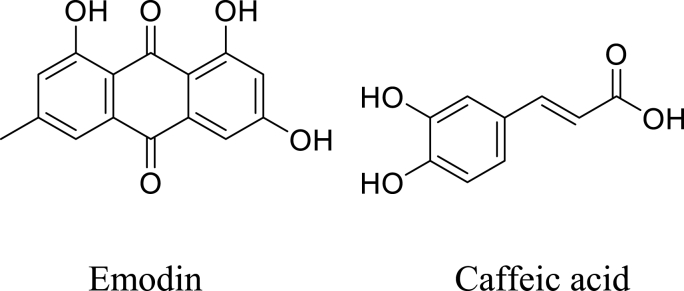
Natural-based compounds that perform as antagonists of ACE2.

Because the patterns of ACE and ACE2 are so similar, compounds that suppress ACE may also reduce ACE2, resulting in a reduction in the viral entrance. Nevertheless, more research is needed to confirm or refute this notion. Patten et al. looked at medicinal herbs to see if they have any anti-ACE2 properties. They discovered 141 therapeutic species from 73 families, as well as 49 pure natural substances with known ACE inhibitor properties [42]. Furthermore, 16 therapeutic species were discovered to be capable of inhibiting ACE II in vitro. *In vitro* discovered four Iranian therapeutic species capable of inhibiting more than 80% of ACE action. *Berberis integerrima* Bunge, *Onopordum acanthium* L., *Crataegus laevigata* (Poir.) DC. and *Quercus infectoria* G. Olivier were the active species. *Quercus infectoria* G. Olivier. was determined to become more energetic at 330 μg/ml, inhibiting ACE by 94%. Its greater phenolic content and antioxidant capacity may account for its significant antagonistic activity. Even though *Q. infectoria* extract has significant ACE suppression and antioxidant properties, the existence of condensed tannins reduced its efficacy by interfering with ACE functions. Without the existence of *C. microphylla*, tannins, *B. integerrima*, and *O. acanthium* are reported as having strong ACE antagonistic effects as well as increased antioxidant capacity. These organisms might be good sources of antiviral compounds. Virus infections do cause oxidative stress, which encourages virus multiplication. Antioxidant species reduce the group of reactive oxygen species (ROS) in infected cells and target several oxidative stress-linked pathways, resulting in a decline in viral propagation. SARS-CoV-2 and SARS-CoV attach to ACE2 by identical affinities. Another investigation discovered that 25 Chinese herbal groups strongly inhibited the SARS-CoV–ACE2 relationship. Polygonaceae, Labiatae, Oleaceae, Magnoliaceae, Lauraceae, and Nelumbonaceae species were found to have the strongest inhibitory effects. Emodin (1,3,8-trihydroxy-6-methylanthraquinone), as shown in Fig. 2, which is generated in excessive quantities in the genus Rheum and Polygonum, was found to be the cause of these inhibitory actions. Emodin, with an IC_50_ of 200 μM, repressed the association between S protein and ACE2 in a dosage-based way [22].

### 3.3 Role of NPs in targeting TMPRSS2

**Fig. 3:**
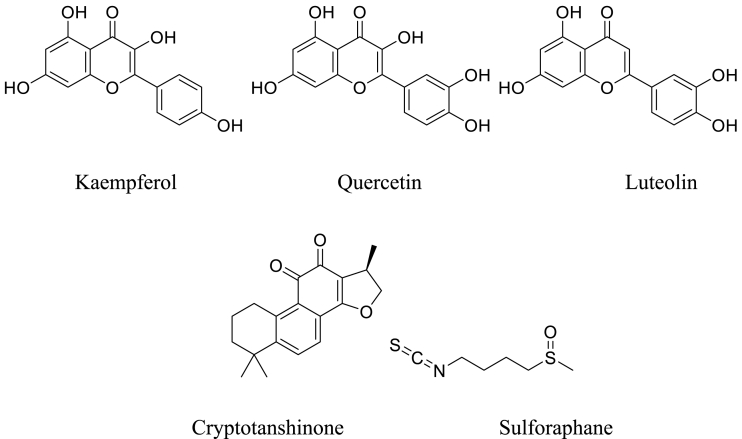
Natural-based compounds that perform as antagonists against TMPRSS2.

The serine-type 2 RSS2 type II transmembrane serine protease, which breaks down proteins the S spike receptors of SARS-CoV and MERS, and ACE2, is a type II transmembrane serine protease. Hoffmann et al. revealed the usage of ACE2 to penetrate host cells; coronavirus also utilizes TMPRSS2 for S proteins priming. The complex is broken mostly by TMPRSS2 after the S spike protein interacts with the ACE2 (host cell) to allow viral entry [40]. Matsuyama et al. discovered that cells with high TMPRSS2 expression are more vulnerable to corona. Because the virus's entry was regulated by its linking to the ACE2, which is cleared by the TMPRSS, discovering drugs that can inhibit or down-regulation TMPRSS2 activity in human cells can be a viable preventive or therapeutic strategy [43]. Numerous studies have shown that natural items can repress or downregulate TMPRSS2. At 5 and 15 mM, kaempferol was found to be capable of inactivating TMPRSS2 transcription at about 49.14 and 79.48%, respectively. Similarly, sulforaphane was discovered to inhibit TMPRSS2 expression by releasing and translocating Nrf 2 (nuclear factor (erythroid-derived 2)-like 2) receptor. TMPRSS2 expression was considerably decreased by a standardized flavonoids preparation containing luteolin, quercetin, and kaempferol. Despite the fact that the three flavonoids have a variety of biological impacts, this analysis revealed that they had a significant synergic activity at lower concentrations. The protection and efficacy of these medicines in COVID-19 patients, however, are still unknown. Furthermore, the clinical utility of such compositions and chemicals may be limited by ways of delivery, the condition of the individuals' digestive system, as well as the stage of disease [44]. A study found that cryptotanshinone, at a concentration of 0.5 μM, has *anti*-TMPRSS2 action was, exemplified in Fig. 3.

### 3.4 Role of NPs in directing PL^pro^

The SARS-CoV-2 genome encodes PL^pro^, which is a nonstructural protein. Because it supports the breakdown of viral polyproteins (PP1A and B) to effector proteins, this protease is essential for virus assembly [45]. PL^pro^ has also been discovered as an opponent of the host's immune function. PL^pro^ has been demonstrated to impede nuclear translocation, IRF3 phosphorylation, dimerization, as well as the NF-kB signaling pathways (by inhibiting IkBa destruction. The Toll-like receptor 3 and retinoic acid-inducible gene-one pathway were found to have these impacts. Furthermore, SARS-CoV PL^pro^ was shown to suppress the TLR7 pathway by inactivating the TRAF3 or 6-TBK1-IRF3 or NF-kB or AP1 signal transduction pathway.

**Fig. 4:**
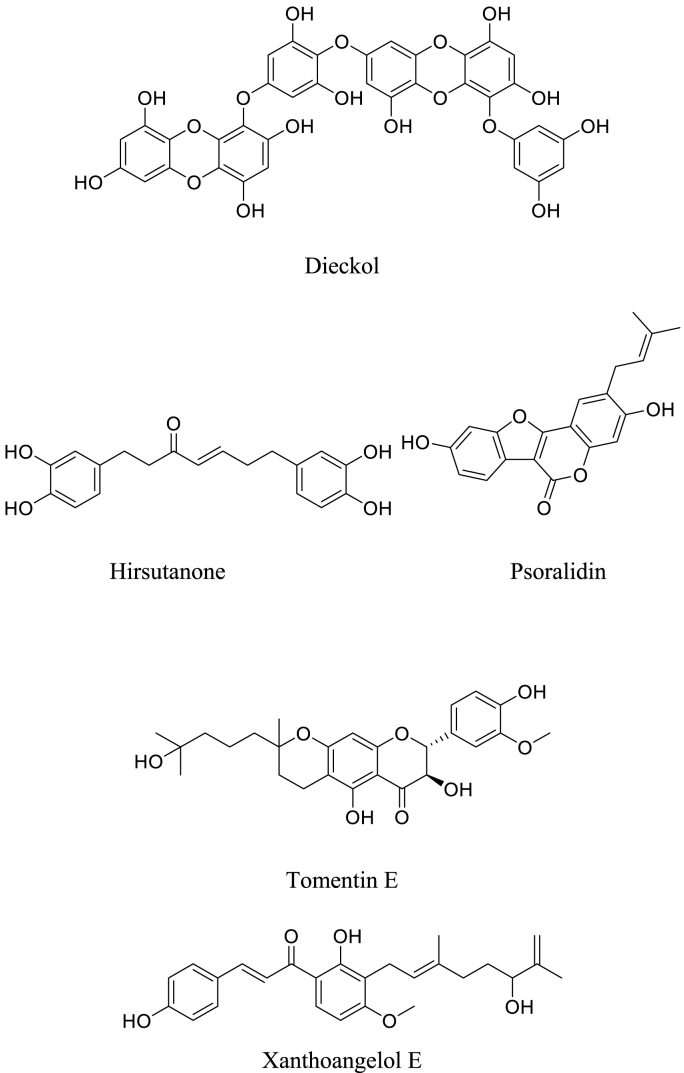
Natural compounds that work as antagonists of PL^pro^.

Arya et al. mainly investigated FDA-ratified medicines for PL^pro^ in silico repressive potential. They found that 16FDA-ratified medicines (Biltricide, Procainamide, Cinacalcet, Terbinafine, Labetalol, Pethidine, Tetrahydrozoline, Ethoheptazine, Ticlopidine, Chloroquine, Levamisole, Naphazoline, Formoterol, Amitriptyline, Benzylpenicillin, and Chlorothiazide) bind to SARS-CoV-2 [46]. A study discovered that Disulfiram (an alcohol-aversive medication) is a competitive antagonist of SARS-CoV PL^pro^. Numerous compounds were revealed to aim at the SARS-CoV PL^pro^ (Fig. 4).

#### 3.4.1 *Tribulusterrestris -* cinnamic amides

Many natural substances have been discovered to have significant PL^pro^ inhibiting properties. Similarly, found that six cinnamic amides secluded from *Tribulus terrestris* (N-trans-Feruloyloctopamine, -Coumaroyltyramine, -Caffeoyltryamine, N-trans-Feruloyltryamine, Terrestrimine, and Terrestriamide) repressed SARS-CoV PL^pro^ in a dosage. The inhibiting IC50 of PL^pro^ in these substances with a molecular weight of 15.8–70.1 μM was discovered. Terrestrimine [(E)-N-(1-hydroxy-2-(4-hydroxyphenyl)-2-oxoethyl)-3-(4-hydroxyphenyl)-3-methoxypheny) acrylamide] was shown to be the most effective antagonist. SARS-CoV PL^pro^ activity, by an IC_50_ of 15.8 0.6 μM. On the one hand, the existence of polar substituent, on the other hand, methylene groups were linked to improved performance action that inhibited [20].

#### 3.4.2 *Cullen corylifolium*– flavonoids

SARS-CoV PL^pro^ was significantly inhibited by an ethanolic excerpt of *Cullen corylifolium* seeds, by an IC_50_ of 15 μg/ml. In addition, six flavonoids included namely, (40 –O-methylbavachalcone, psoralidin, Bavachinin, isobavachalcone, neobavaisoflavone, and corylifol A) suppressed SARS-CoVPLpro action in a dosage-dependent way, by IC_50_ values ranging from 4.2 to 38.4 μM. The greatest repressive impact was applied through psoralidin and isobavachalcone [17].

#### 3.4.3 *Paulownia tomentosa* – flavonoids

Cho et al. isolated 5 novel geranylated flavonones from the ethanolic of *Paulownia tomentosa* fruits: tomentin A, B, C, D, andE. These flavonoids, along with 7 other knowns, inhibited SARS-CoV PL^pro^ in a dosage-based way by IC_50_ values of 5.0 and 14.4 mM. With an IC_50_ of 5.0 to 0.06 μM, Tomentin E had the strongest suppressing activity. Suppression was shown to be higher in substances with a 3,4-dihydro-2H-pyran group. The flavonoids from P. tomentosa were discovered as reversible combined antagonists [47].

#### 3.4.4 *Angelica keiskei* – chalcones

Nine alkylated chalcones (isobavachalcone, xanthoangelol, xanthokeistal F, D, E, B, G, and A, and 4-hydroxyderricin) and four other coumarins isolated from *Angelica keiskei* ethanolic excerpt. The alkylated chalcones reduced SARS-CoV PL^pro^, according to (Miq.) Koidz in a dosage-dependent way, by IC_50_ varying from 1.2 ± 0.4 to 46.4 ± 7.8 μM. The coumarins studied, on the other hand, had no substantial suppressive activities on SARS-CoV PL^pro^. Isobavachalcone was revealed to be a mixed antagonist, while the other chalcones were non competitive, according to kinetic investigations. In comparison to the other examined chalcones, xanthoangelol E, a –OOH replaced counterpart, demonstrated the greatest improved inhibitory, which is 40-fold greater. In silico investigations revealed that xanthoangelol E has a strong inhibitory effect [23].

#### 3.4.5 *Salvia miltiorrhiza Bunge*–Tanshinones

The ethanolic excerpt of *Salvia miltiorrhiza* Bunge repressed SARS-CoV PL^pro^ by 88%. Seven bio-active tanshinones (tanshinone IIA, IIB, methyl tanshinonate, and others) were also discovered. Tanshinone I, dihydrotanshinone I, and cryptotanshinone, the n-hexane fraction yielded the compounds rosmariquinone and rosmariquinone. These Tanshinones were tested for their capability to suppress SARS-CoV. A fluorometric test was used to measure PLpro activities. Both compounds had potent repressive time-dependent actions, by IC_50_scoresvaried from 0.8 to 30 μM. A structure of dimethyl tetrahydronaphthalen has been linked to increased inhibition activity. Cryptotanshinone was discovered as the most effective suppressor of SARS-CoV PL^pro^, by an IC_50_ of 0.8 ± 0.2 μM. Rosmariquinone was recognized as a mixed-kind antagonist of SARS-CoV PL^pro^ in kinetic studies, while other tanshinones were noncompetitive antagonists [15].

#### 3.4.6 *Alnus japonica* – diarylheptanoids

As from ethanolic extracts of *Alnus japonica*, Park et al. identified nine diarylheptanoids (platyphyllenone, platyphyllone, hirsutanonol, platyphyllonol-5-xylopyranoside, oregoninrubranol, hirsutenone, rubranoside B, and A). They used a continual fluorometric test to assess their SARS-CoV PL^pro^ inhibiting impact. The findings revealed that hirsutenone, oregonin, rubranol, hirsutanonol, rubranoside B, and A had considerable dosage-based repressive effects against hirsutanonol, hirsutenone, rubranol, oregonin, rubranoside B, and A. IC_50_ values for SARS-CoV PL^pro^ range from 3 to 44.5 μM. Hirsutenone have the strongest repressing effects, with an IC_50_ of 4.1 ± 0.3 μM, that was low substantial than curcumin blocker. Because of its increased inhibition activity, the existence of an unsaturated fatty acid appears to be linked to diarylheptanoids groups of carbonyl and catechol [15].

### 3.5 Role of natural products in targeting 3CL^pro^

**Fig. 5:**
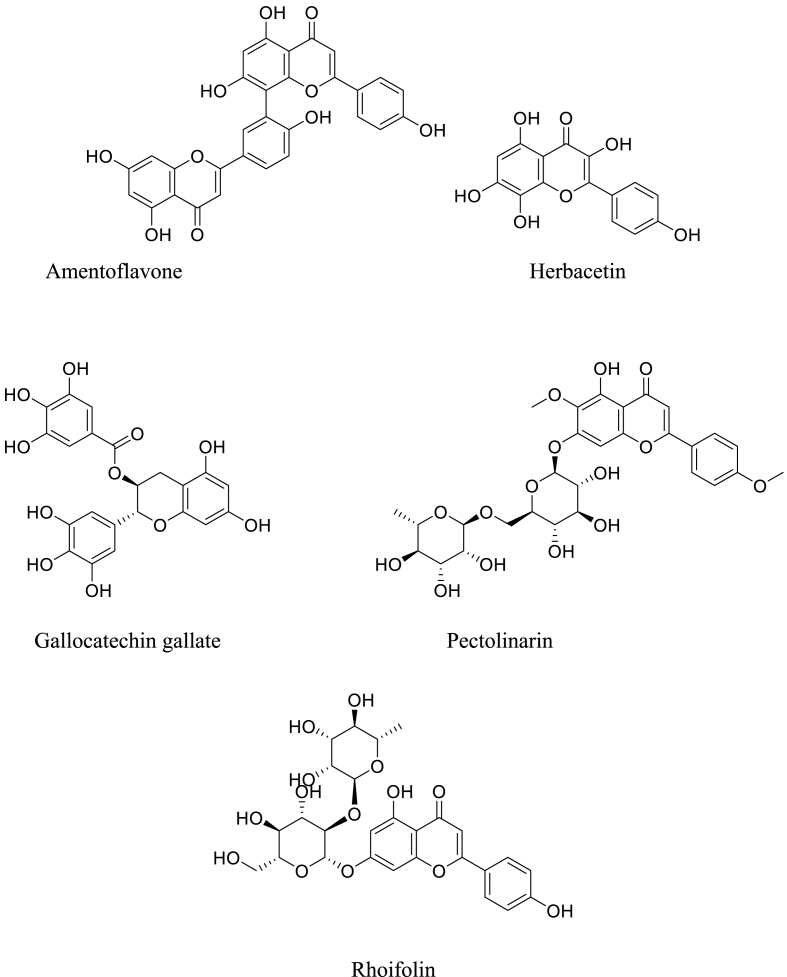
Natural compounds perform as antagonists of 3CL (pro).

3CL (pro) is one of the SARS-CoV-216 nonstructural proteins. 3CL (pro) was a promising beneficial targeting for *anti*-COVID-19 medicines since it shows an important part in corona replicating processes polyproteins. Anti-3CL (pro) activity was demonstrated in a variety of natural substances (Fig. 5).

#### 3.5.1 *Angelica keiskei* - alkylated chalcones

To use a fluorescence resonance energy transfer (FRET) technique, the suppressive efficacy of alkylated chalcones and coumarins isolated from *Angelica keiskei* (Miq.) Koidz towards SARS-CoV-3CL (pro). Alkylated chalcones, except coumarins, showed strong inhibitory activity in a dosage-dependent way. The IC_50_score varies from 11.4 to 1.4 and 129.8–10.3 μM. Xanthoangelol E was also discovered to become the utmost effective SARS-CoV-3CL (pro) antagonist. Both alkylated chalcones were found to be competitive antagonists in kinetic experiments. Because xanthoangelol E inhibits SARS-CoV-PL^pro^, it may be an effective alternative in the COVID-19 treatment protocol. The compound's structures are illustrated in Fig. 5.

#### 3.5.2 *Ecklonia cava* - phlorotannins

From an ethanolic excerpt of brown Algae *Ecklonia cava*, nine phlorotannins. To use a cell-free experiment, the inhibition effect of these phlorotanninson SARS-CoV-3CL (pro) was identified. Eight phlorotannins have been demonstrated as an effective antagonist of SARS-CoV-3CL (pro) in a dosage-dependent way. Diekcolis the most effective antagonist of SARS-CoV-3CL among the compounds examined (pro). Diekcol has the least binding energy in the direction of SARS-CoV-3CL, which was confirmed by molecular docking experiments (pro). Diekcol formed stronger H-bonding with the catalytic dyad, namely, Cysteine 145 and Histidine 41. Nonetheless, phlorotannin availability and inter-individual variability in their metabolism of dietary remain significant limitations invalidating their utility. The structure of the gut microbiota appears to be important in establishing their medical benefits. Furthermore, the variety of their structures adds to the intricacy of their framework's links, as well as structural and conformational differences. Because of the lack of isomers for similar molecular weight, a nonappearance of analytical standards and a clear link here between the two compositions and bioactivity could be another constraint in therapeutic usage [48].

#### 3.5.3 *Salvia miltiorrhizabunge*–tanshinones

The antagonistic efficacy of *Salvia miltiorrhiza* Bunge against SARS-CoV-3CL was examined. They discovered a 30 μg/ml ethanolic extract of *Salvia miltiorrhiza* Bunge inhibited SARS-CoV-3CL by 60%. Also identified, six tanshinones from the plant (lipophilicity portion) inhibited SARS-CoV-3CL (pro) in a dosage-dependent and not the time-dependent way. The IC_50_were calculated to be between 14.4 and 89.1 μM. With just an IC_50_ of 14.4 0.7 μM, dihydrotanshinone I was found to be the most receptor antagonist. *Salvia miltiorrhiza* Bunge tanshinones were discovered as non-competitive antagonists of SARS-CoV-3CL (pro) in terms of kinetic mechanisms.

#### 3.5.4 *Torreya nucifera* - biflavonoids

To use the FRET technique, 4biflavonoids were extracted from the ethanol part of *Torreya nucifera* & Zucc. and assessed for its 3CL (pro) antagonistic activity. SARS-CoV-3CL (pro) was inhibited through all biflavonoids, by IC_50_ values varying from 8.3 to 72.3 μM. The cytotoxic activity of 8 diterpenoids taken out from T. nucifera extract was higher. Because it had the smallest IC_50_ (8.3 1.2 μM), amentoflavone had the most potent depressive effect. Furthermore, it had a greater inhibition activity than apigenin, quercetin, and luteolin. Amentoflavone has a higher affinity for SARS-CoV-3CL (pro) and established stronger H-bonding. A stronger inhibition activity is thought to be owing to its apigenin component at position C-30 of flavones [49].

#### 3.5.5 Flavonoids

The cytotoxic effect of 7 flavonoids on SARS-CoV-3CL (pro) produced in *Pichia pastoris* GS115 was investigated. Quercetin Gallocatechin gallate and epi-gallocatechin gallate, all inhibited 3CL (pro) action through 91, 85, and 82%, respectively, at a level of 200 μM. With an IC_50_ of roughly 47 μM, gallocatechin gallate was discovered as a competitive antagonist of SARS-CoV-3CL (pro).

**Fig. 6:**
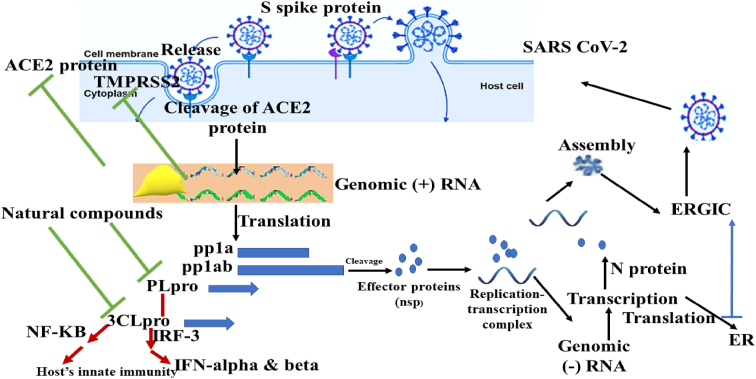
Overview of probable *anti*-COVID-19 activities of organic compounds.

Because of the hydrophobic and H-bond interactions created by the catalytic spot of SARS-CoV-3CL (pro), docking experimentations verified gallocatechingallate's strong inhibitory. Nonetheless, Chen et al. found it challenging to foresee how these H-bonds would relate to the biochemical processes of the ostensibly bioactive components. Moreover, the intensity of the H-bonds is not studied, and the results may restrict the specificity of the examined molecules because a significant number of weaker H-bonds might enhance affinity and hence interactions with non-targets. Fig. 6 outlines the probable anti-corona activities of organic products [50].

### 3.6 Promising NPs towards SARS-CoV-2

**Fig. 7:**
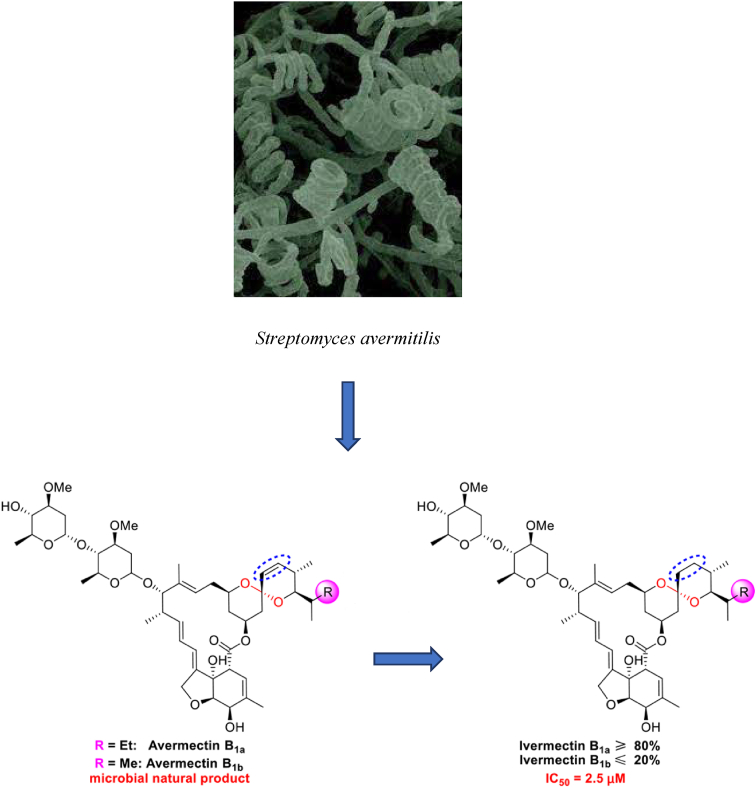
Auspicious natural-substance-inspired ivermectin for the action of COVID-19.

In 1978, Mura's group collaborated with Merck Sharp and Dohme Research Laboratories to produce ivermectin, a groundbreaking broad-spectrum anti-parasitic medication. It was shown to be highly successful as an oral medicine for treating head lice (94.9% effectiveness at 24 h and 73.8% efficiency at two weeks. Ivermectin, a semi-synthetic 22,23-dihydro derivative of avermectin B1a and b, is a naturally occurring substance generated by *Streptomyces avermitilis* (Fig. 7). Ivermectin is a commonly utilized antibiotic in humans and animals.

Ivermectin's usage is presently being broadened. Wagstaff's team, for example, found that ivermectin was particularly active in controlling corona RNA production in vitro, resulting in a 5000-fold decrease in the virus after 48 h. Lohmer's team recently noticed that one oral dosage of ivermectin was improbable to achieve the IC_50_ (2.5 mM) in the lungs (anticipated lung concentration: 0.0857 mM), and those pharmacological treatments or ingested therapeutic interventions (to raise the density of in the lungs) could be regarded as possible solutions. Nonetheless, its security in humans has been repeatedly demonstrated, and this is envisaged it will become a crucial factor for future medical treatment plans for COVID-19 [51].

Gilead Sciences’ C-nucleoside analog GS-5734 (remdesivir), a wide antiviral substance, showed encouraging preclinical effects in the therapy of HIV, the first instance of corona outbreaks in the United States. Onsynthesizing GS-5734 (a prodrug) was the subject of the first investigation, tubercidin (an antibiotic) was structurally modified*. Streptomyces tubercidicus* produces an adenosine analog by substituting a C–C bond for the C–N connection to get 4-aza-7,9-dideazaadenosine.4-aza-7,9-dideazaadenosine has the same effectiveness as tubercidin towards HL-60 cells and is more hydrolytic stable [52].

Nevertheless, 1′-CN partially replaced Nuc, perceived as a novel framework inspired by natural cyanide toyocamycin (secluded from *Streptomyces toyocmnsis*), which has significantly contributed to antiviral drug design. *In vitro*, Nuc suppresses the hepatitis C virus substantially. GS-441524 has indeed been observed as the activated state of Nuc in disease cells, although the monophosphate transformation of Nuc to GS-441524 a rate-limiting process. To get around this, the monophosphorylated prodrug GS-5734 was created to address this problem to improve cell viability in vivo.

GS-5734 have considered a capable broad-spectrum antiviral medication that was found to have active toward a diversity of viruses, notably SARS- and MERS-CoV. Moreover, GS-5734 was unearthed as extremely active toward SARS-CoV-2 infections in vitro throughXiao's team and had lower toxicity. In terms of modes of action, Li's team discovered that GS-5734 could connect to corona RdRp RNA-binding channels. The 1′-ribose CN change found in GS-5734 is critical for blocking SARS-CoV-2 viral RNA transcription [53].

Since 2016, GS-5734 has indeed been shown to be effective and safe for EBOV illness and SARS-CoV-2 infections. On April 10, 2020, Gilead Sciences announced that 68% of individuals with symptomatic corona who have been addressed by GS-5734 improved clinically, with no novel safety concerns discovered. In particular, the US FDA granted GS-5734 an urgent use authorization (EUA) in May 2020 for the therapy of coronavirus. To enhance the effectiveness of drug design at GS-5734, scientists at Gilead Sciences created a scalable GS-synthesis technique. There is currently no drug–free options available for data on GS-5734 medication interactions, but there's a chance of clinically serious side effects interaction rates is low. Its true GS-5734 is expected to be validated as a secure and reliable medicine for SARS-CoV-2 [54].

Palmer's team at Emory University found the N-nucleoside analog EIDD-2801, a potential vocally accessible antiviral drug. The first step in the synthesis process is to conduct some preliminary investigation. The development of EIDD-2801 started with structural changes to the broad-spectrum receptor. N4-deoxycytidine is an antiviral agent which has been generated from natural essential oil. Uridine is a substance occurring in healthy plasma [54].

Lately, Emory University scientists found a scalable method for manufacturing EIDD-2801. The usage of EIDD- 2801, a potential antiviral drug, is received considerable attention a lot of attention. EIDD-1931, is particularly successful at reducing SARS-CoV-2 reproduction, according to Baric's team. In Calu-3 cells and Vero cells, EIDD-1931 showed significant *anti*-SARS-CoV-2 action with no cytotoxic effects at dosages up to 5 mM. EIDD-2801 was also an orally accessible prodrug, which was effectively digested in vivo, and has a broad therapeutic window [55].

EIDD-2801 also enhanced the pulmonary role of infected mice by MERS-CoV or SARS-CoV, according to Baric's team. Although there is a dearth of convincing evidence in humans, EIDD-2801 has shown potential usefulness. It is intended that scientists would try to tackle the obstacles (like manufacturing on a large scale, effectiveness, and security in vivo) so that clinical studies can be conducted [55].

### 3.7 Other small compounds by in vitro action towards SARS-CoV-2

The usage of small compounds has been recognized as a major prospective approach for addressing COVID-19 as our knowledge of effective antiviral medication discovery has grown. The organ selenium molecule ebselen, as well as other antagonists of corona major protease (M^pro^) revealed strong activity, having IC_50_ values in the micro- or sub-micromolar range, according to Rao's team. Ebselen inhibits SARS-CoV-2 infection with strong potency and is moderately toxic. Most significantly, its security in humans has been repeatedly assessed in numerous clinical investigations. Many other NPs and NP-inspired prospective small compounds, in addition to the aforementioned small compounds, have revealed significant *anti*-SARS-CoV-2 action [6].

Drug discovery is fraught with dangers, even though the ratified protease inhibition lopinavir and ritonavirare assumed as highly efficacious towards COVID-19 (as they were mentioned as active towards SARS, Wang's team found that lopinavir coupled with ritonavir doesn't appear to be remarkably efficient in COVID-19 individuals.

Due to its possible to efficiently suppress SARS-CoV-2, chloroquine, and hydroxychloroquine have received much interest. For instance, Xiao's team demonstrated that chloroquine effectively prevented SARSCoV-2 infections in vitro at a lower dose. Even though the US FDA ratified chloroquine and hydroxychloroquine for usage in coronavirus individuals, the WHO has declared that medical evidence does not support their usage in these individuals. Furthermore, chloroquine can be fatal in youngsters (limited therapeutic windows), thus, it should be administered with caution [56].

### 3.8 Metabolites from natural resources towards coronavirus

The generation of coronavirus vaccines involves several problems, including ensuring that they are safe and efficacious, as well as manufacturing and distribution. As a result, in pandemic containment, supportive therapies were more feasible in a shorter period. The mainstays of action are symptom management and virus replication suppression. Additionally, transmission can be limited through societal measures [57].

The sensitivity for the virus rather than the host metabolic, in other words, developing an effective therapy with lower toxicity, is a difficulty in the creation of antiviral medication, including synthetic medicines. In this connection, the pharmaceutical sector is increasingly turning to organic antiviral medications [58].

The specificity of any antiviral therapy includes synthetic medications against the virus rather than the host metabolic is a problem in the growth of any antiviral therapy and includes synthetic medications. Treatment that is both effective and minimal in toxicity. In this sense, the pharmaceutical industry plays a significant role. The industry is increasingly turning to organic antiviral compounds with activity [58].

Since the epidemic in 2003, various natural resources from the plant have indeed been evaluated for *anti*-SARS-CoV efficacy and utilized as a framework in medication production (Table 3). Avones, fatty acids, avonols, terpenes, alkaloids, and tannins, are among the organic metabolites. The diverse mechanisms employed through every phytochemical group's ability to impedeCOVID-19 account for the variety of these classes of organic compounds. Nevertheless, the chemical compositions of these natural compounds share common characteristics that support those who found that SARS-CoV suppression needs chemical assemblies with a hydrophobic, OH groups, aromatic ring, and carbohydrates moiety based on in silico research. While not all *anti*-SARS-CoV substances contain an aromatic ring, they all feature lipophilic and hydrophilic areas, as well as the potential to make numerous hydrogen bonds via hydroxyl groups.

**Table 3:** Antivirus natural metabolites confirmed in vitro*.*

SL. No.	Natural source	Compound name	Method used	Ref.
1.	Black tea	Tannic acid	Fluorogenic substrate peptide	[60]
2.	*Lactuca sativa*	Leukamenin	Not informed	[61]
3.	*Artemisia annua*	Ethanol extract	MTS assay	[62]
4.	*Pyrrosia lingua*	Chloroform extract	MTS assay	
5.	*Strobilanthescusia*	Tryptanthrin	MTT assay	[63]
6.	*Stephania tetrandra*	Fangchinoline	MRC-5 system	[64]
7.	*Stephania tetrandra*	Cepharanthine	MRC-5 system	
8.	*Mycale* sp.	Mycalamide A	Not informed	[65]
9.	*Euphorbia neriifolia*	Ethanolic extract of Friedelin	MRC-5 system	[66]
10.	*Euphorbia neriifolia*	Ethanolic extract of Friedelanol	MRC-5 system	

Many natural metabolites that are *anti*-SARS-CoV also exhibit bioactive effects towards other virus kinds or illnesses. The maritime sponge mycalamide A as well as its equivalent, mycalamide B, for example, both have bioactivity toward Herpes virus. Conversely, myricetin, an avonol, exhibits antiviral properties forleukaemia, HIV as well as the influenza virus.Lycorine, is also renowned for a wider variety of pharmacologic applications, like antioxidant, antimicrobial, antitumoral, anti-inflammatory, and cytotoxic activities [59].

As seen in the fight against SARS-CoV, natural products offer a great deal of potential in coronavirus therapy. Wen et al. discovered the natural metabolites ferruginol (A), 8b-hydroxyabiet-9 (11),13-dien-12-one (B), 7b-hydroxydeoxycryptojaponol (C), 3b, 12-diacetoxyabiet-6,8,11,13-tetraene (D), betunolic acid (E), and savinin (F) showed IC50 values ranging between 0.63 (betunolic acid) to 1.57 mM (3b, 12-diacetoxyabiet-6,8,11,13-tetraene).In spite of the fact that these values were greater than those of a manufactured medicine, they show that natural compounds have amazing bioactivity. Bioproducts that contain those metabolites also weren't created to act as *anti*-coronaviruses. However, they are frequently exploited and ingested by their lower IC50 values in people, which shows that SARS-CoV therapy is effective and can be evaluated in a long-term manner. Furthermore, the assessment of their inhibiting action and chemical structure deliver fresh and unique drug development frameworks comprising four abietane-type diterpenes (A–D), one triterpene, and lignan (F) are among the metabolites [67].

## 4 Conclusions

Remdesivir, favipiravir, lopinavir, ritonavir, and arbidol are some of the antiviral therapy options that have been found as prospective alternatives to the medicine that is currently used to treat COVID-19. These drugs work through a variety of different methods of action, including the prevention of virus replication within host cells and the inhibition of viral entry into host cells [[68], [69], [70], [71]]. In addition, immunotherapeutic approaches have emerged as one of the most prominent therapeutic modalities in recent years. Anakinra, sarilumab, siltuximab, and tocilizumab are a few of the immunomodulatory and anti-inflammatory medications that have been proposed as potential treatments against COVID-19 [69,[72], [73], [74]]. The use of convalescent plasma treatment and monoclonal antibodies has been characterized as a potential therapeutic strategy that is both effective and reliable [69,73]. This method seeks to strengthen patients' immune systems and prevent them from contracting viral infection [[69], [70], [71], [72], [73], [74]]. Nevertheless, medicinal plants and NPs are still seen as viable options for preventing and treating various ailments. Because the COVID-19 disease broke out in December 2019, various traditional herb medications were used with excellent results among COVID-19 patients, primarily in China. We addressed the possible applications of therapeutic herbs and normal items to protect or perhaps cure COVID-19 in this review. Even though studies assessing the antiviral impacts of therapeutic herbs were quite inadequate and undeveloped, some NPs with IC50 values under ten μM might be regarded as potential *anti*-SARS-CoV-2 substances because they are capable of preventing SARS-CoV-2 life-cycle binding proteins corresponding to cell protein ACE2, papain-, or chymotrypsin-like proteinases. However, various issues have been identified in terms of the specificity of the actions performed through such items, maintainable procurement of species, dose ranges utilized, and the application of adequate controls. However, there are various indicators that plant-based medicines might aid in the battle against COVID-19 disease; more studies are required to determine the medical utility of such compounds for COVID-19 infection. The antiviral benefits of herbal mixes, therapeutic plants, or natural items must be studied in observational and experimental research. Aside from the necessity for scientific authentication of their protection profile, the biological availability of natural compounds by potential antivirus properties, like tannins, must be studied.

## Ethical approval

This article does not require any human/animal subjects to acquire such approval.

## Sources of funding

This study received no specific grant from any funding agency in the public, commercial, or not-for-profit sectors.

## Author contribution

Kannan Raman: Conceptualization, Data curation, Writing-Original draft preparation, Writing- Reviewing and Editing. Kalirajan Rajagopal: Conceptualization, Data curation, Writing-Original draft preparation, Writing- Reviewing and Editing. Fahadul Islam: Data curation, Writing-Original draft preparation, Writing- Reviewing and Editing. Manish Dhawan: Data curation, Writing-Original draft preparation, Writing- Reviewing and Editing. Saikat Mitra: Data curation, Writing-Original draft preparation, Writing- Reviewing and Editing. Baliwada Aparna: Data curation, Writing-Original draft preparation, Writing- Reviewing and Editing. Potlapati Varakumar: Data curation, Writing-Original draft preparation, Writing- Reviewing and Editing. Gowramma Byran: Data curation, Writing-Original draft preparation, Writing- Reviewing and Editing. Om Prakash Choudhary: Data curation, Writing- Reviewing and Editing. Talha Bin Emran: Conceptualization, Writing-Reviewing and Editing, Visualization.

## Trial registry number

1. Name of the registry: Not applicable.
2. Unique Identifying number or registration ID:Not applicable
3. Hyperlink to your specific registration (must be publicly accessible and will be checked): Not applicable.

## Guarantor

Talha Bin Emran, Ph.D., Associate Professor, Department of Pharmacy, BGC Trust University Bangladesh, Chittagong 4381, Bangladesh. T: +88-030-3356,193, Fax: +88-031-2550224, Cell: +88–01819942214. https://orcid.org/0000-0003-3188-2272. E-mail: talhabmb@bgctub.ac.bd.

## Consent

Not applicable.

## Data availability statement

The data that support the findings of this study are available from the corresponding author upon reasonable request.

## Declaration of competing interest

The authors declare that they have no conflict of interest to disclose.
